# Targeting the stem cell niche micro-environment as therapeutic strategies in aging

**DOI:** 10.3389/fcell.2023.1162136

**Published:** 2023-05-19

**Authors:** Raheleh Farahzadi, Behnaz Valipour, Soheila Montazersaheb, Ezzatollah Fathi

**Affiliations:** ^1^ Hematology and Oncology Research Center, Tabriz University of Medical Sciences, Tabriz, Iran; ^2^ Department of Anatomical Sciences, Sarab Faculty of Medical Sciences, Sarab, Iran; ^3^ Molecular Medicine Research Center, Tabriz University of Medical Sciences, Tabriz, Iran; ^4^ Department of Clinical Sciences, Faculty of Veterinary Medicine, University of Tabriz, Tabriz, Iran

**Keywords:** Adult stem cells, stem cell niche, aging, regenerative medicine, cell transplantation

## Abstract

Adult stem cells (ASCs) reside throughout the body and support various tissue. Owing to their self-renewal capacity and differentiation potential, ASCs have the potential to be used in regenerative medicine. Their survival, quiescence, and activation are influenced by specific signals within their microenvironment or niche. In better words, the stem cell function is significantly influenced by various extrinsic signals derived from the niche. The stem cell niche is a complex and dynamic network surrounding stem cells that plays a crucial role in maintaining stemness. Studies on stem cell niche have suggested that aged niche contributes to the decline in stem cell function. Notably, functional loss of stem cells is highly associated with aging and age-related disorders. The stem cell niche is comprised of complex interactions between multiple cell types. Over the years, essential aspects of the stem cell niche have been revealed, including cell-cell contact, extracellular matrix interaction, soluble signaling factors, and biochemical and biophysical signals. Any alteration in the stem cell niche causes cell damage and affects the regenerative properties of the stem cells. A pristine stem cell niche might be essential for the proper functioning of stem cells and the maintenance of tissue homeostasis. In this regard, niche-targeted interventions may alleviate problems associated with aging in stem cell behavior. The purpose of this perspective is to discuss recent findings in the field of stem cell aging, heterogeneity of stem cell niches, and impact of age-related changes on stem cell behavior. We further focused on how the niche affects stem cells in homeostasis, aging, and the progression of malignant diseases. Finally, we detail the therapeutic strategies for tissue repair, with a particular emphasis on aging.

## Background

Adult stem cells (ASCs) are undifferentiated cells with the ability of self-renewal and differentiation into specific cell types in the adult body. Some adult tissues contain ASCs capable of producing differentiated cells that are appropriate for their locations. In other words, ACSs, as tissue stem cells, can repair and maintain tissue hemostasis through self-renewal and differentiation into distinct and specialized cell types. For instance, ASCs that reside in the central nervous system (CNS) can generate neurons, astrocytes, and oligodendrocytes. These tissue-resident stem cells play an essential role in the growth and regeneration of tissues under normal physiological conditions and following injury. ASCs are found in a variety of adult tissues and can be divided into mesenchymal stem cells (MSCs), hematopoietic stem cells (HSCs), neural stem cells (NSCs), liver stem cells (LSCs), induced pluripotent stem cells (iPSCs), pancreatic stem cells (PSCs) and the like, etc. (Clevers, 2015; Chowdhury and Ghosh, 2021). MSCs are commonly used in scientific research because of their wide range of sources, including bone marrow (BM), muscle, fat, and amniotic fluid. MSCs are a type of ASCs with a high level of self-renewal and multi-differentiation ability. These cells are ideal for tissue engineering, regenerative applications, and the treatment of various disorders (Fitzsimmons et al., 2018). ASCs share a variety of common characteristics. Many ASCs undergo asymmetric cell division to produce two daughter cells: one represents stem cell identity, and the other enters the differentiation path necessary for tissue homeostasis, development, and regeneration. In other words, an imbalance between differentiation and self-renewal dynamics can cause tissue degeneration, and cancer (Zion et al., 2020). ASCs also express telomerase for self-renewal and to maintain replicative capacity. ASCs persist mainly in a quiescent state, which is crucial for self-renewal potential. Despite their quiescent state, ASCs have the intrinsic potential to gain replication competence to regenerate specific tissues in response to injury or stress. This metabolic plasticity can switch between the stem cell phenotype and differentiated phenotype (Cianflone et al., 2019).

**FIGURE 1:**
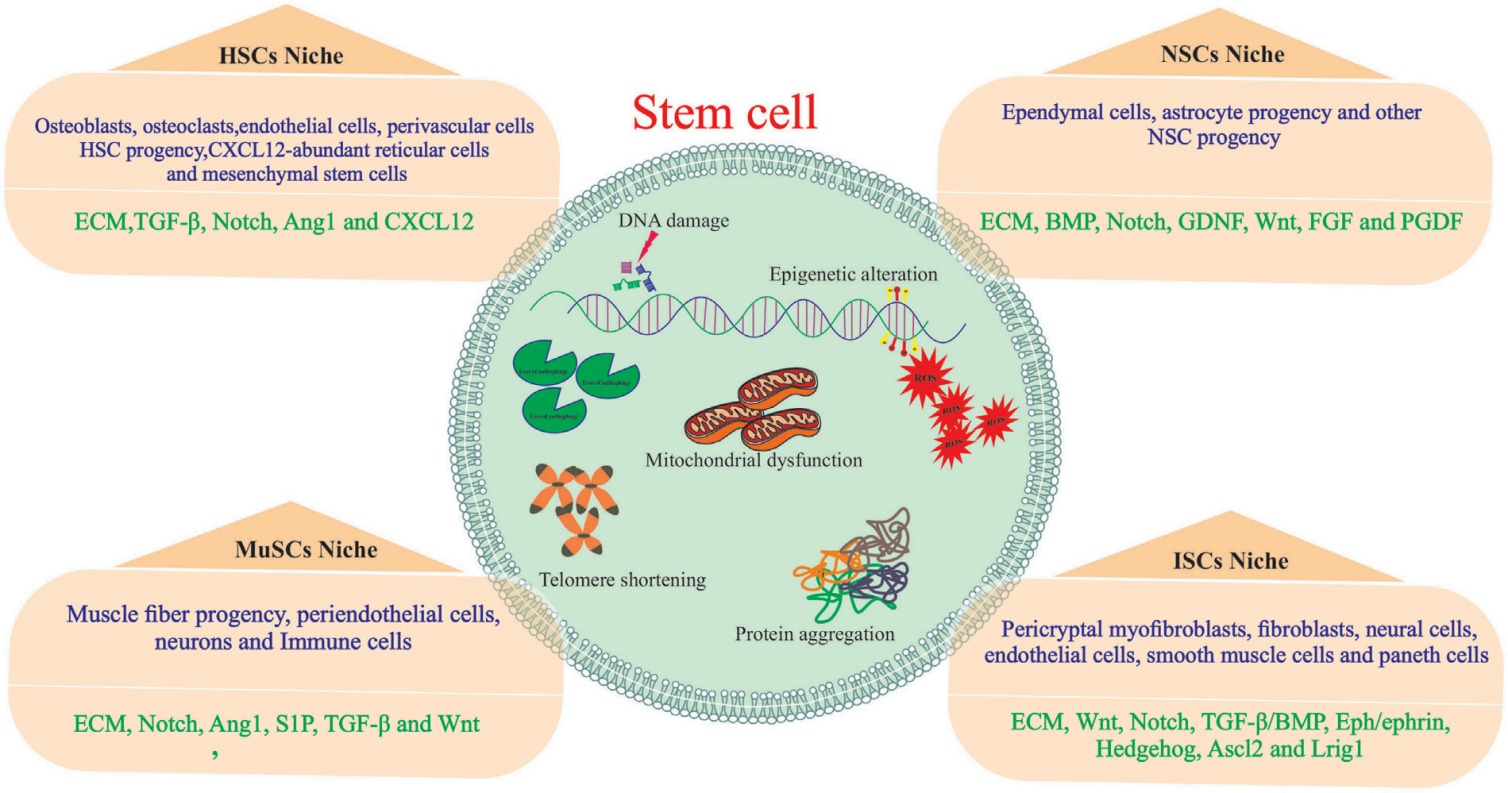
Major influences of stem cell aging. Stem cells are influenced by both intrinsic and influences within them and extrinsic influences from the stem cell niche. The stem cell niche is composed of both cellular (blue) and acellular (green) components.

Cellular senescence is a fundamental aging mechanism, defined by irreversible and stable cell cycle arrest upon exposure to a variety of stressors. Aging contributes to a number of diseases, including cancer and cardiovascular diseases. Aging, as an inevitable change, is manifested by the accumulation of DNA damage, disturbances in protein homeostasis, disruption of cellular communication, and stem cell exhaustion (Sameri et al., 2020). Generally, when cells reach their Hayflick limitation after a number of divisions, they begin to age and ultimately die by apoptosis. ASCs play a crucial role in maintaining tissue regeneration and hemostasis by replenishing dying cells (Di Micco et al., 2021); however, ASCs undergo senescence and apoptosis during aging. Senescence-specific phenotypes of ASCs are observed during culture, such as reduced cell proliferation, morphological changes, and imbalanced biological activities. With this view, a better understanding of stem cell aging is necessary for *in vitro* expansion and subsequent employment in basic and clinical settings, providing insight into organ aging (Schultz and Sinclair, 2016; Liu et al., 2020). Aging affects stem cell features in terms of self-renewal ability, differentiation potential, and regenerative capacity. Stem cell function is controlled by both intrinsic (cellular genetic factors) and extrinsic (environmental stimulation) factors (Ermolaeva et al., 2018). Changes caused by intrinsic mechanisms include DNA damage, imperfect protein homeostasis, mitochondrial dysfunction, accumulation of reactive oxygen species (ROS), and epigenetic reprogramming. Cell-extrinsic factors arise from alterations in the niche, depletion of stem cell pools, and several local and systemic factors, which can considerably influence the functions of stem and progenitor cells and an organism’s lifespan. Collectively, ASCs aging is driven by cell-intrinsic and cell-extrinsic alterations (Oh et al., 2014; Schüler et al., 2020). A hazardous alteration in extrinsic or intrinsic factors reduces stem cell number, affecting the regenerative potential of stem cells As a result, age-related diseases are more likely to develop due to age-associated changes in intrinsic and extrinsic factors (Figure 1) (Maharajan et al., 2020).

Relying on this evidence, modulating the stem cell niche may contribute to preserving and restoring the youthful characteristics of stem cells and promoting health during aging (Pennings et al., 2018).

Stem cell niche is a paracellular microenvironment that plays a substantial role in maintaining the principal properties of stem cells. This microenvironment possesses cellular and non-cellular compositions from both systemic and local sources. The constituents of the niche differ in various tissues; however, the coordination of stem cell functions in all tissues is similar. Importantly, the aging of the stem cell microenvironment can significantly affect stem cell function (Szabó et al., 2022).

Several studies using allogeneic transplants have shown that exogenous factors affect the aging of NSCs, germline stem cells (GSCs), and satellite cells (Rožman et al., 2018). Stem cells and their niches provide tissues with plasticity that enables them to adapt to local or systemic variations (Rojas-Ríos and González-Reyes, 2014). To preserve ASCs function, it is crucial to understand the mechanisms and signals involved in the regulation of stem cell behavior. As mentioned, the niche’s cellular and acellular composition provides structural support and signals, either locally or systemically, for the normal functioning of stem cells. Together, age-related alterations in stem cell niches and their components may lead to maladaptive functional changes in stem cells and the loss of tissue omeostasis (Comazzetto et al., 2021). This review discusses the role of stem cell niches in stem cells aging. We also summarized niche-targeting strategies for future therapeutic applications in stem cell transplantation and regenerative medicine.

### Stem cells niches

**FIGURE 2:**
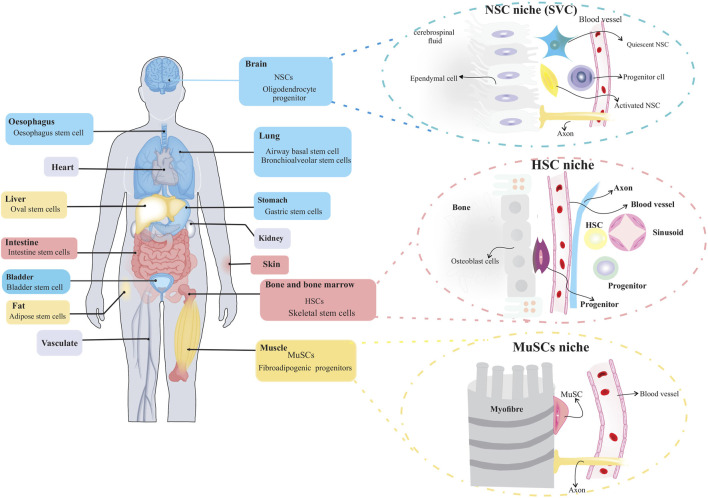
Organs with stem cells and stem cell niches. Several organs in humans and other mammals contain stem cells and stem cell niches. The figure depicts organization of adult stem cell niches in the brain, blood and muscle.

**FIGURE 3:**
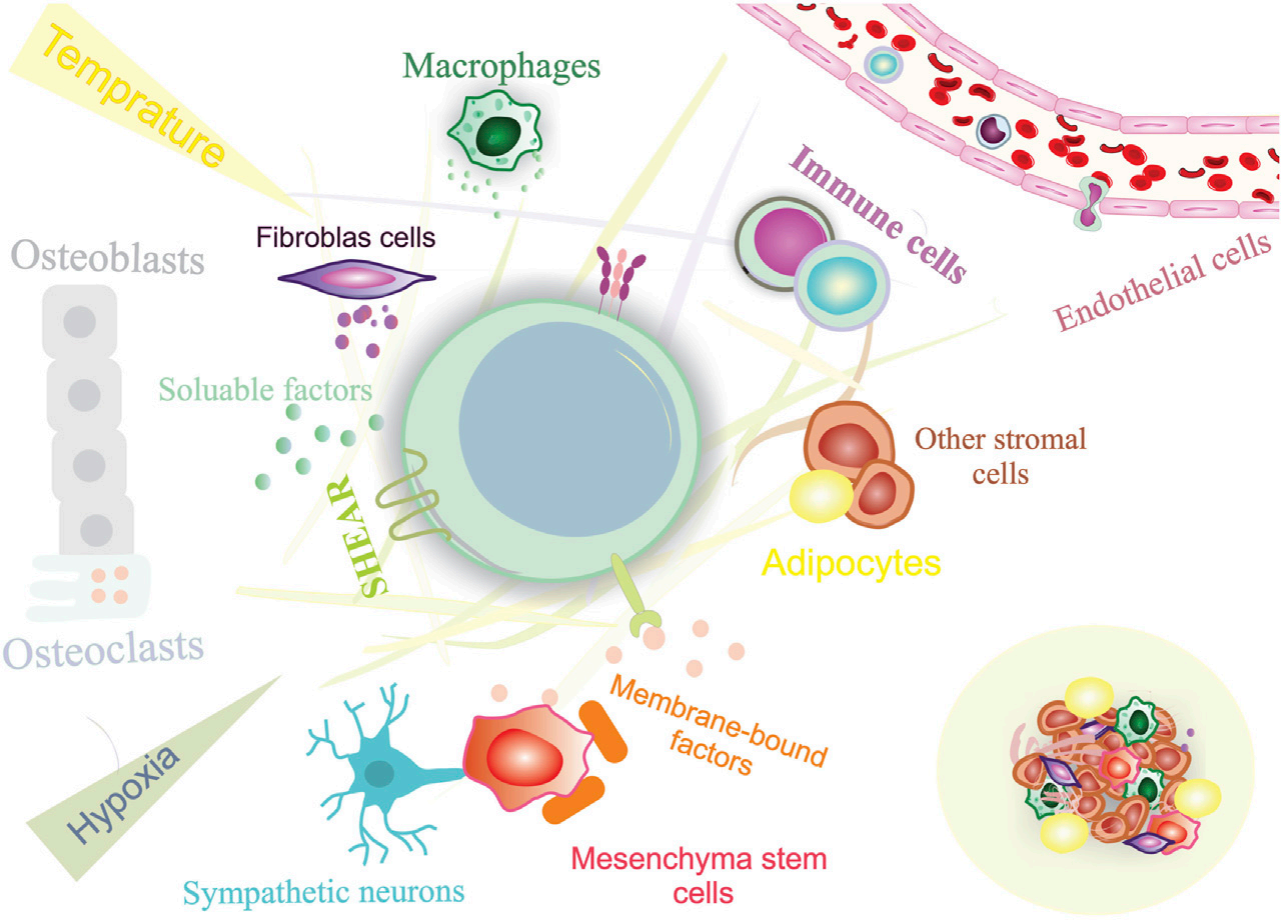
Constituents of a stem cell niche. Stem cell niches are highly complex, including both cellular and acellular components.

Stem cells play an essential role in the growth and regeneration of many tissues; thereby, they are critical for maintaining normal tissue function. It is, therefore, imperative to protect stem cells from damage or loss while maintaining sufficient interaction with their environment at the same time to respond to physiological signals for cell replacement and repair. This balance between stem cell protection and interaction appears to be achieved by preserving stem cells within specialized niches (Gomariz et al., 2020; Ottoboni et al., 2020). The stem cell niche is a spatial organization that provides anatomical and functional interactions that are critical for stem cell fate. In fact, the niche protects and coordinates stem cell function in a temporal and spatial context. Several tissues have been identified as stem cell niches, such as the bone marrow, germline skeletal muscle, digestive and respiratory systems, mammary glands, and central and peripheral nervous systems (Figure 2) (Brunet et al., 2022). A niche consists of stromal cells as well as the factors they produce, such as adhesive molecules, soluble factors, and matrix proteins. Numerous laboratories have elucidated the key constituent of stem cell niches that include specific cell types such as mesenchymal, neuronal/glial, vascular and immune/inflammatory cells, adhesion molecules, and surface-associated signaling receptors, as well as physical factors such as shear stress, matrix rigidity, oxygen pressure, and temperature. The stem cell niche is a specialized and dynamic microenvironment that provides survival and proper stem cell function (Figure 3) (Wagers, 2012; Ceafalan et al., 2018).

Within the niche, cellular contact with surrounding cells and adjacent stem cells is responsible for structural support, adhesive interactions, and the release of soluble signals that control stem cell function (Pennings et al., 2018). Extracellular matrix (ECM) interactions can generate retention signals and mechanical cues to retain stem cells in the niche, allowing them to respond to external stimuli (Ahmed and Ffrench-Constant, 2016). The ECM is a dynamic, tissue-specific environment that regulates cell behavior. This network is also used for the proper function, development, and repair of particular organs through remodeling of its components (Gattazzo et al., 2014; Novoseletskaya et al., 2019). Stem cells interact directly with ECM proteins such as integrins, laminin, fibronectin, tenascin-C, and collagen VI (Sonbol, 2018). Integrins and laminins are the most critical ECM proteins that regulate stem cell function and behavior. ECM can also sequester and concentrate growth factors, chemokines, and other stem cell-related factors by binding locally and systemically to the elements produced (Theocharis et al., 2016; Birbrair, 2017). Several stem cell types are closely associated with the vasculature and the nervous system. This can modulate stem cell responsiveness to metabolic signals and circadian rhythms, providing a conduit to deliver immune cells and inflammatory/humoral factors into the niche (Fielding and Méndez-Ferrer, 2020).

Furthermore, stem cell responses to external stimuli are also influenced by physical factors such as shear forces, temperature, and chemical signals provided by the niche. It is important to note that the components of the stem cell niche may differ between diverse types of tissue and under different physiological conditions; however, all signals generated by these components determine stem cell fate in terms of self-renewal, differentiation, retention/migration, reversible switch between proliferation and quiescence and survival/death. In other words, niche factors activate specific intracellular signaling pathways to direct the fate of stem cells. Despite having the same basic constituents, stem cell niches in different organs exert different signaling pathways to regulate stem cell function (Chacón-Martínez et al., 2018; Singh et al., 2019). The pivotal role of stem cell niche in the proper functioning of stem cells offers a powerful opportunity to manipulate stem cells to enhance their therapeutic efficacy. Indeed, as revealed by definition, stem cell niches extrinsically impact stem cells’ function; thereby, these anatomical networks can be considered as a novel “druggable’’ target for regenerative applications rather than stem cells. Accordingly, niche targeting therapies could be of interest to improve stem cell functionality in transplantation through endogenous targeting strategies or model systems *in vitro* (Bardelli and Moccetti, 2017).

Given the importance of stem cell-based therapeutic applications, investigating stem cell niches can help to remodel the microenvironment to expand stem cells *in vitro*. Therefore, the following paragraphs aim to shed light on the relationship of stem cells with their niche and related components and consider the advancement, challenges, and opportunities of stem cell niche engineering to employ stem cell-based therapies.

### The cellular components of the niche

The cellular components of stem cell niche can be categorized into the stem cells and their progeny, neighboring mesenchymal stromal cells, distant cells such as neurons and endothelial cells, immune cells, and ECM, as a mechanical structure.

Components that are conserved in the niche include the following (Ferraro et al., 2010).

1. Stromal supportive agents such as cell adhesion molecules and soluble secreted factors found within the stromal niche in proximity to the stem cells.
2. The ECM acts as an anchor for stem cells and serves as a mechanical scaffold for stem cell signal transmission.
3. A blood vessel that delivers nutrients and signals from the niche to other organs, and participates in recruiting stem cells from and to the stem cell niche
4. Inputs from the nervous system promote the mobilization of stem cells from their niches and facilitate the integration of signals from various organs. Neuronal cues appear to play a significant role in the trafficking of HSCs (Katayama et al., 2006).

Niche resident cells regulate the functionality of stem cells, including those that physically bind to stem cells such as stromal supporting cells and mesenchymal cells, and those that provide paracrine signals. Overall, supportive cells and other niche cells, known as resident niche cells, contribute to the proper functioning of stem cells in adult tissues (Wagers, 2012).

**FIGURE 4:**
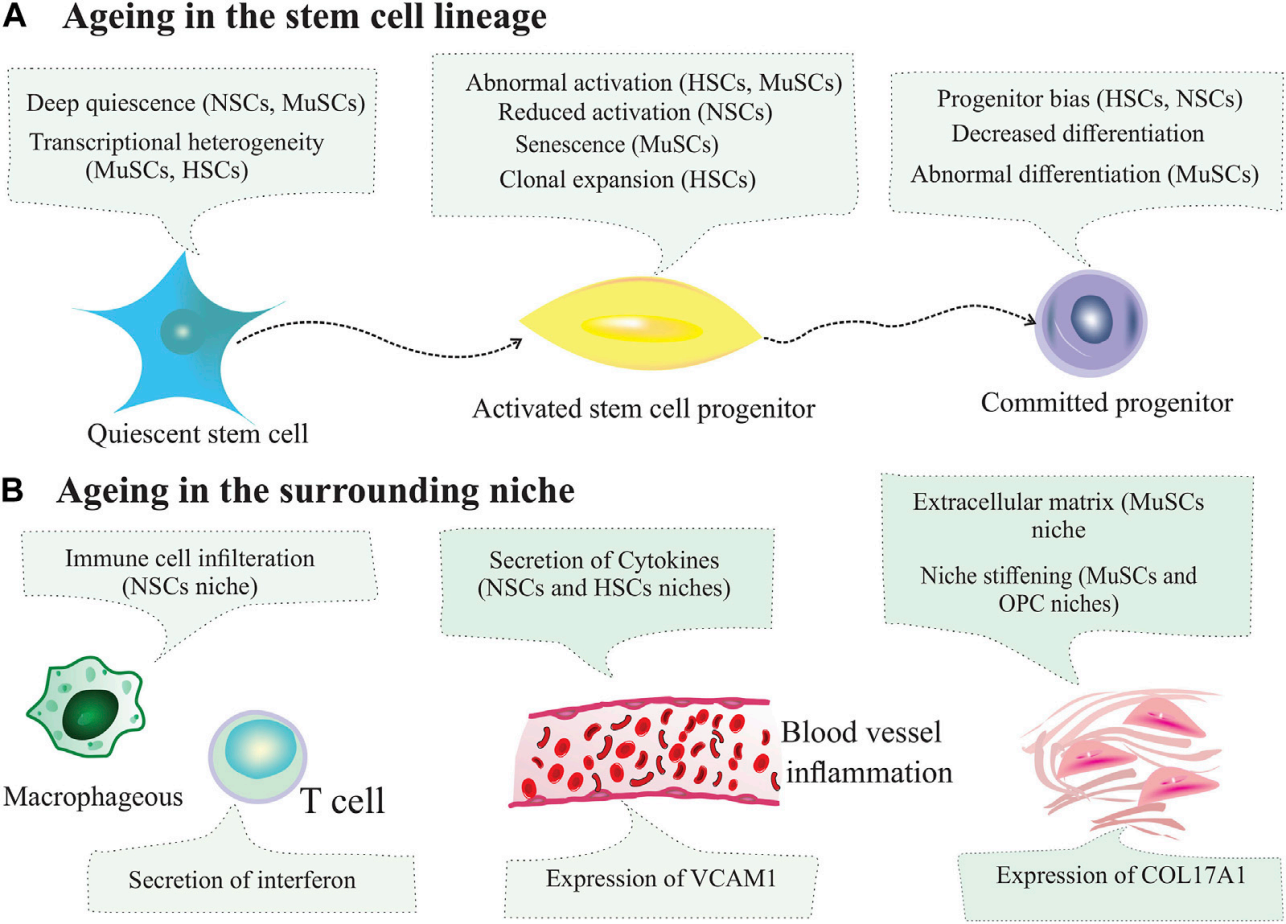
Changes in stem cells and their niches during ageing. **(A)** Ageing changes inthe stem cell lineage. During ageing, subpopulations of stem cells can enter deep quiescence, and their ability to activate is reduced. Other subpopulations of stem cells can exhibit aberrant activation, reduced activation and clonal expansion. **(B)** Ageing changes in the niche. Ageing leads to immune cell infiltration, inflammation, secretion of cytokines, expression of cell adhesion molecules by blood vessel cells, extracellular matrix secretion and increased stiffening of the niche.

The hematopoietic system maintains the homeostatic levels of all blood lineages, including red blood cells, myeloid cells, lymphoid cells, and platelets. The vast majority of these cells are produced in the BM of multipotent HSCs (Montazersaheb et al., 2022a). In a steady-state status, the hemapoietic system is maintained by HSCs, which reside in the BM niche (Farahzadi et al., 2023). In other words, BM niche cells support HSCs. HSCs are mainly dormant and rarely enter the cell cycle; however, they can exit a quiescent state in response to various stimuli. The self-renewal capacity of HSCS is maintained by intrinsic and extrinsic factors from the local and systemic environments. Within this niche, BM stromal cells regulate HSCs function, including MSCs, osteolineage, and endothelial cells. Niche cells provide physical support and regulate the HSC homeostasis. In this complex niche, interactions between cellular components depend on the molecular stimuli. Niche function and composition can be altered by physiological conditions and aging (Figure 4) (Asada et al., 2017; Brunet et al., 2022). HSCs reside in the BM to produce all the cells necessary for the immune system and blood replenishment by self-renewal and differentiate into their progenies. Intrinsic and extrinsic signals from the niche tightly regulate the homeostasis of the hematopoietic system. According to the identification methods by Wei et al., different BM niche cells outnumber the number of HSCs in the BM at least 20 times (Wei and Frenette, 2018). For instance, HSCs reside and are tightly supported by the perivascular network and cell populations composed of MSCs, endothelial cells, bone-lining osteoblasts, monocytes, macrophages, and megakaryocytes (Sugiyama et al., 2019). The endosteal niche plays a critical role in the maintenance of HSCs because HSCs are present in the endosteal niche at the endosteum. The essential components of this niche are macrophages, endothelial cells, and MSCs surrounding blood vessels. In this regard, the loss of BM macrophages, such as osteoblast-supportive endosteal macrophages, can suppress the expression of HSC-supportive cytokines in the endosteum, eliciting HSCs mobilization from the niche into the bloodstream. In addition, depletion of endosteal osteoblasts results in defective endosteal bone formation and reduced levels of factors required to retain the self-renewal ability of HSCs (Winkler et al., 2010). Aging contributes to the stem cell niche remodeling. During aging, HSC-supporting niches (endosteal BM) are reduced, and non-endosteal BM expands further from the bone (Montazersaheb et al., 2022b). Although HSCs residing in the BM accumulate with age, they are functionally impaired. Aged HSCs are associated with increased self-renewal ability, decreased potential to differentiate into the lymphoid lineage and display dominance of the myeloid lineage. It has been reported that aged mice exhibited reduced endosteal niches with fewer osteoprogenitor cells (Ho et al., 2019). Many changes have been observed in HSCs during aging, including reduced regenerative capacity, ROS accumulation, DNA damage, myeloid skewing, and metabolic alterations (Broxmeyer et al., 2020). All of these alterations significantly affected the size of the HSCs pool. Despite the expansion of HSCs and megakaryocytes during aging, these cells are located farther from each other within the niche, indicating the impact of megakaryocytes on HSCs and age-related complications (Ghosh et al., 2021).

Germline stem cells (GSCs) are located in a defined niche regulated by various factors to maintain the balance between the self-renewal and differentiation of GSCs. GSCs in *Drosophila* are a model for investigating the regulation of ASCs *in vivo*. *Drosophila* is a valuable animal model for studying aging. There is an age-dependent alteration in the GSCs niche in *Drosophila,* that affects its regenerative ability. The male and female gonads of *Drosophila* are well-characterized anatomical niches in which stem cell signaling and stem cell-niche interactions are elucidated. The ovary of *Drosophila* is a valuable tool for investigating the mechanism of aging owing to the presence of maternal GSCs and somatic follicle stem cells. Aging can reduce fertility in many species, including humans (Piper and Partridge, 2018). Ishibashi et al. used the *Drosophila* ovary as an injury model of aging. Utilizing this model could provide a reliable system for studying the effects of aging on the division and differentiation of stem cells *in vivo*, similar to mammalian stem cell niches (Ishibashi et al., 2020).

Moreover, utilizing the testes of aged *Drosophila* revealed that GSCs maintenance is mediated by secreted factors from the testicular niche which is composed of diverse somatic cells. An imbalance between the self-renewal and differentiation of GSCs could impede reproduction, causing germ cell loss and infertility (Chang et al., 2019). Age-related changes in the GSC niche significantly affect *Drosophila* regenerative capacity. GSCs reside within a niche of somatic cap cells and hub cells; therefore, loss of these cells in the ovary and testes causes alterations in the GSCs populations in aged *Drosophila* (Kahney et al., 2019). Kai et al. reported that an empty niche in *Drosophila* ovarioles might maintain tissue homeostasis under particular conditions. As an example, the loss of niche GSCs is perfectly compensated by reactivating the ectopic cell proliferation, therefore, empty niches can persist and tissues can remain in a homeostatic state (Kai and Spradling, 2003). Under conditions of stem cell ablation, the niche can establish a favorable microenvironment for somatic cells to replicate and compensate for the loss of stem cells, and maintain tissue homeostasis (Maharajan et al., 2020).

NSCs are another example of coordination between niche cells and stem cells during tissue hemostasis. NSCs are undifferentiated neural cells characterized by extensive replication capacity, long-term self-renewal, and potential to differentiate into neuronal and glial cells in the CNS. Unlike CNS cells, NSCs can remain dormant for long periods of time and provide a reserve pool of cells for tissue regeneration and cell replacement throughout their lifespan. The cellular components of NSC niches, their responses to local or systemic signals, and their physical properties (e.g., stiffness) are altered during aging. Although all cells in the brain are affected during aging, NSCs in the adult brain can generate neurons (neurogenesis) and regenerate some brain functions (Andreotti et al., 2019). The mammalian brain has a few niches for NSCs: the subgranular zone (SGZ) and the subventricular zone (SVZ). The latter is a well-characterized stem cell niche. These regions support neurogenesis throughout developmental stages, diseases, injuries, and old age. NSC isolated from the SVZ can differentiate into neurons and glia *in vitro* (Ruddy and Morshead, 2018). CNS injury is accompanied by the scar tissue formation. As a result of scar formation in the injured spinal cord, secondary damage is reduced due to mechanical stability and restriction of inflammatory cell infiltration.

On the other hand, scar formation can inhibit the growth of injured axons. Resident NSCs give rise to new astrocytes that form glial scars at the injured site in the spinal cord. Indeed, NSC-derived astrocytes contribute to the restriction of lesion enlargement after injury.

Furthermore, NSCs progeny have a neurotrophic effect that is required for the survival of neurons adjacent to the lesion. Collectively, stem cell transplantation or NSCs-derived cells lead to functional recovery of the injured spinal cord. The interplay between the NSCs and their niche has a significant impact on neurogenesis. A better understanding of niche alterations during aging may help develop novel therapeutics and interventions to improve neural repair (Sabelström et al., 2013).

### Acellular/molecular regulation of stem cell niche

As outlined above, stem cell niches contain both cellular and acellular components. The stem cell niche is a dynamic microenvironment that supports stem cell functionality. Multiple mechanisms contribute to the crosstalk between stem cells and the niche, including soluble factors, adhesion molecules, and many signaling pathways such as Wnt and Notch. In addition to the cellular components within the niche, acellular components are also involved in directing stem cell fate. In the case of HSCs, quiescent HSCs reside within the niche and exit, upon activation. As an adhesion molecule, E-selectin is exclusively expressed by the BM endothelial cells in the vascular niche. E-selectin expression in the vascular niche maintains quiescent HSCs. This molecule is crucial in regulating HSC and their proliferation; hence, E-selectin is regarded as an essential component of the vascular niche (Fielding and Méndez-Ferrer, 2022). E-selectin knockout mice or administration of E-selectin antagonists causes an increase in the quiescence and self-renewal potential of HSCs. Winkler et al. showed that E-selectin blockade could enhance HSC proliferation and survival by up to three to six folds in mice treated with chemotherapeutics or radiotherapy. Since high-dose chemotherapy causes severe BM suppression, a transient blockade of E-selectin may be a promising approach for protecting HSCs during chemotherapy or radiation therapy (Winkler et al., 2012).

Stem cells and niche resident cells can communicate with each other by secreting signaling molecules and recognizing them *via* membrane receptors. In addition, stem cell number remain constant in many tissues; however, age-related functional decline in stem cells may lead to stem cell aging and senescence in diverse types of tissues. Prolonged activating of quiescent stem cells in tissue depletes quiescent stem cells and their regeneration ability. With this notion, stem cells are maintained within the niche by several essential factors and signals (Crane et al., 2017). Acellular components, such as ECM and signaling factors, contribute to the maintenance of stem cells during their lifespan. Single-cell analysis revealed that ECM components regulate stem cell function and behavior (Watt and Huck, 2013). There is considerable variation in the ECM components and physical characteristics (topography and strength) of the various niches. ECM components are critical regulators of downstream signaling pathways.

ECM proteins include laminin, collagen, integrin, and fibronectin (Gattazzo et al., 2014). The integrin family consists of heterodimeric transmembrane receptors that mediate adhesion and promote stem cell-niche interactions. These surface adhesion receptors mediate crucial intracellular signal transduction related to cellular phenotypes such as adhesion, proliferation, differentiation, migration, and survival (Isomursu et al., 2019). Integrins are important regulators of various physiological and pathological conditions, including development, aging, and cancer. In this regard, abnormal integrin signaling is involved in many age-related diseases. It is believed that integrin-mediated cell adhesion contributes to apoptosis induction by modulating the intrinsic (mitochondrial) and extrinsic (death receptor) pathways, which decide whether a cell lives or dies (Borghesan and O'Loghlen, 2017; Humphries et al., 2019). As illustrated in the previous sections, aging is affected by intrinsic and extrinsic pathways, which exhibit crosstalk to determine stem cell fate. The dynamic interplay among intrinsic, environmental, altered stem cell niche, and systemic factors contributes to the functional loss of stem cells during aging. Aging can be influenced by local signals and systemic factors (developmental changes, circadian rhythms, and metabolic activity). These factors reduce stem cells’ proliferative and regenerative capacity, affecting homeostasis (Rojas-Ríos and González-Reyes, 2014; Ermolaeva et al., 2018). Accumulating evidence indicates that stemness is a dynamic state that is governed by several factors within a niche. Stem cell niches are composed of multiple secretory and soluble factors. In this context, stem cells respond to various environmental signals and fulfill the needs of the tissues. Several signals are involved in this event, including secreted signals from the stem cells, adjacent niche cells, or other tissues. Notch signaling, signaling from adhesion receptors within the ECM, and mechanical signals can also provide key signals to stem cells (Chacón-Martínez et al., 2018).

In the case of the intestinal stem cell niche, communication between intestinal stem cells (ISCs) and their environment is regulated by several signaling pathways such as Notch, Wnt/*β*-catenin, Hedgehog, and TGF-*β*/BMP pathways. Disturbance of these pathways or ECM composition can considerably influence the ISC niche, causing an increasing the likelihood of intestinal diseases (Meran et al., 2017).

As previously discussed, NSCs reside within specific niches and differentiate into neurons, astrocytes, and oligodendrocytes. The NSC niche is a dynamic environment close to the cerebrospinal fluid (CSF) and systemic circulation. Thus, NSCs respond not only to local microenvironment factors, but also to those that exist in the systemic macroenvironment. These factors can be soluble molecules, such as cytokines and chemokines from circulation, or local factors released by microglia and astrocytes. The activity of NSCs depends on local signals, systemic factors (e.g., blood and CSF), immune cells, and CNS resident cells such as neurons, astrocytes, and microglia. In the context of aging, systemic regulation becomes even more evident since significant changes in circulating cytokines and chemokines greatly impact the functions of NSCs (Willis et al., 2022).

Muscle stem cells (satellite cells or MuSCs or SCs) are the long-term ASCs that are esponsible for the regeneration of skeletal muscles. Under homeostastatic condition, MuSCs are quiescent. A significant alteration occurs in niche components upon muscle injury, exposing MuSCs to signaling cues to repair damaged tissue (Kann et al., 2021). An age-related decline in the regenerative potential of skeletal muscles is associated with MuSC dysfunction. In other words, the repair of skeletal muscle depends on MuSC function. Upon injury, MuSCs are activated and proliferate to form myofibers. In aged muscle, there is a shift from repair cascades toward increased ECM deposition, which can be correlated with the dysfunction of MuSC proliferation. The skeletal muscle ECM plays a crucial role in tissue structure by force transmission and elastic response. Age-related changes in ECM architecture within skeletal muscle show that aging is highly associated with the ECM’s pathogenic phenotype, increased muscle stiffness, and decreased collagen tortuosity. In aged MuSCs, myogenesis decreases and fibrogenesis increases (Stearns‐Reider et al., 2017). ECM and its components can collectively provide space between cells and regulate many aspects of stem cell behavior (Nicolas et al., 2020).

It has been shown that exposure to circulating factors from the blood of young-aged animals caused the rejuvenation of aged MuSC with the restoration of muscle regeneration. A young host provides a favorable environment and generates a youthful phenotype from transplanted aged cells (Rando and Jones, 2021). A heterochronic transplantation study between young and old mice revealed a restoration of the proliferative and regenerative potential of MuSCs in aged mice by exposing them to the sera from young mice. It appears that systemic factors from young mice can trigger MuSC proliferation in old mice and prevent age-related decline (Conboy et al., 2005). It has been reported that Notch signaling in satellite cells is reduced with age and impacts the regenerative potential of old skeletal muscle. However, it has been found that restoration of the systemic factors in old mice can reactivate Notch signaling and promote satellite cell proliferation (Sueda and Kageyama, 2020). Pax7-expressing muscle cells are capable of self-renewal and repair of skeletal muscles. In aged skeletal muscles, a noticeable decline in Pax7-expressing muscle cells was observed under homeostatic conditions. The mechanism behind this decline is attributed to age-associated niche changes. The stem cell niche is an essential factor in the maintenance of quiescence, a state that preserves the number and function of ASCs. The close association of MuSCs with mature muscle fiberes is important for maintaining a quiescent state during homeostatic conditions. Accordingly, muscle fibers are crucial components of the muscle niche. Aged dormant MuSCs strongly express sprouty 1 (Spry1), inhibiting fibroblast growth factor (FGF) signaling. Under homeostatic conditions, a higher level of FGF2 signaling in aged MuSCs with the removal of Spry1 led to the loss of quiescent stem cells, MuSC depletion, and a decline in regenerative capacity (Chakkalakal et al., 2012).

Spermatogonial stem cells (SSCs) are male GSCs that support gamete production by self-renewal and differentiation into daughter cells. The maintenance of SSC functionality unequivocally depends on the testis niche. With advancing age, dysfunction or loss of SSCs occurs. Transplantation of SSCs into aged mice showed that stem cells alone might not be able to change their functions. Instead, the niche influences the balance between self-renewal and differentiation capabilities. In this regard, SSCs transplantation of an aged mouse into a young recipient showed continual spermatogenesis over 3 years (Ryu et al., 2006). This observation explains why the stem cell niche in young animals is essential for the homeostasis and regeneration of stem cells. The exhaustion of stem cells in the stem cell pool is believed to result in an imbalance between quiescence and proliferation of stem cells (Chakkalakal et al., 2012).

Cadherins are functional membrane proteins that contribute to cell-cell adhesion. E-cadherin, an essential adhesion factor, is responsible for providing physical support and the maintaining GSC in *Drosophila*. An age-dependent decline in E-cadherin expression in the GSC niche leads to reduced GSCs in male and female *Drosophila* (Kahney et al., 2019; Pascal et al., 2021). Overall, homeostasis is governed by the dynamic activity of resident stem cells and niche composition. These factors can affect stem cell aging. Therefore, treatments targeting niche composition and dynamics may provide therapeutic benefits.

### Aging of the bone marrow vascular niche

Significant morphological and metabolic alterations occur in the BM endothelium with age.

Imaging and flow cytometric analyses revealed an overall reduction in the arteriolar proportion. The significant decrease in arteriolar vessels and perivascular stromal cells that express platelet-derived growth factor receptor beta (PDGFR-β) is associated with a decreased level of stem cell factor (SCF) in aged mouse long bones. Conversely, the abundance of sinusoidal type L blood vessels remains unchanged during aging. There is a decline in type H vessel density, accompanied by a decrease in blood flow to the BM, which may elicit metabolic changes in the BM. The decline in type H vessels with age is correlated with a decrease in osteogenesis, osteoprogenitors, bone density, and endosteum niches (Ho et al., 2019; Stucker et al., 2020).

The aged BM endothelium also exhibits other metabolic changes, such as increased hypoxia and ROS levels. These changes were associated with decreased angiogenesis and migration capacity. Furthermore, aging reduces endothelial expression of hypoxia-inducible factor 1 (HIF-1), which is responsible for age-related changes and loss of bone density and type H endothelium. Compared to young mice, aged BM ECs express lower levels of prohematopoietic factors. Several factors contribute to HSC homeostasis including SCF, CXCL12, and Notch ligands. Additionally, type H ECs and adjacent perivascular cells exhibited higher Notch signaling activity. Indeed, there is a link between age-related decreases in endosteal vessels and impairment in endothelial Notch activity, together with a decline in type H vessels. In line with this, activation of the endothelial Notch signaling pathway enhances bone blood flow and the abundance of HSCs. In addition to functional and metabolic alterations, the aged endothelium also exhibits remarkable morphological changes, such as vascular leakage, vasodilation, and impaired vessel integrity. During aging, perivascular cells also undergo functional changes, and with age, the abundance of pericytes decreases, leading to a reduction in quiescence factors, such as SCF, Bmp4 and Bmp6. These events contribute to the loss of quiescence of HSCs and cancer cells (Ho et al., 2019; Sivan et al., 2019).

Additionally, aging may increase HSC numbers by reducing perivascular secretion of Osteopontin. BM MSCs have a reduced proliferation potential and are biased toward adipogenesis at the expense of osteogenic differentiation. Myeloid skewing and impaired functionality of aged HSCs may be due to age-related accumulation of adipocytes in the BM, which inhibits HSC function and B lymphomagenesis. Given that adipocytes have the potential to inhibit HSC functionality and B-lymphomagenesis, age-related accumulation of adipocytes in the BM may stimulate myeloid skewing and impair the function of aged HSCs. Although the number of HSCs increases with age, their function decreases, showing a reduction in self-renewal and loss of quiescence. It is likely that these age-related alterations are associated with an HSC shift from endosteal arteriolar niches to non-endosteal sinusoidal BM niches. Several cell-intrinsic dysfunctions, including protein misfolding and DNA damage accumulation, have been implicated in age-related changes in HSCs. A growing body of evidence indicates that changes in the BM niche contribute to the age-related reduction of HSC function. It has been established that the co-culture of young HSCs with aged ECs and *in vivo* infusion of aged ECs following myelosuppression inhibits repopulation capacity and is linked to myeloid lineage bias in the hematopoietic system. Infusion of young ECs significantly enhances repopulation potential and partially restores HSC function, indicating a bidirectional interplay between HSC aging and alterations of the vascular niche. Hematopoietic aging decreases immune system functionality. A compromised immune system increases the likelihood of infection, autoimmune diseases, and hematological malignancies, which further alters the vascular niche and interferes with HSC homeostasis (Poulos et al., 2017).

### Targeting the niche can improve stem cell transplantation

In regenerative medicine, one of the most widely celebrated successes is the clinical application of BM transplantation to regenerate the blood-forming system (Fathi et al., 2022). Typically, BM transplantation involves harvesting HSCs from the marrow cavity of the ilium or peripheral blood (after treatment with agents that induce migration of HSCs into the circulation) and infusing the cells to a recipient displaying a defect in blood-forming cells or ablated with radiation or chemotherapy. Hence, one of the primary challenges in BM transplantation is attaining sufficient regeneration in a short time period to be effective. This requires collecting an adequate number of stem cells, intravenous injection, migration into the appropriate BM niche, and proliferation and replenishment of lost cells. In other words, successful hematopoietic recovery depends on the efficient homing and engraftment of HSCs into specific BM niches. An ineffective or inefficient performance during these steps may result in graft failure, a life-threatening complication (Szade et al., 2018; Booth, 2022). Strategies should be developed to increase the regeneration rate, reduce transplant-associated complications, and enhance engraftment success. This can be achieved by supplying more regenerative cells or increasing the output of the transferred cells. Notably, strategies targeting stem cell niches may be effective in both approaches. The stem cell niche provides a specialized and regulated environment that determines stem cell fate (Nakamura-Ishizu et al., 2020). Therefore, targeting the stem cell niche has already proven effective in BM transplantation, where donors are administered granulocyte colony-stimulating factor (G-CSF) or plerixafor (AMD3100) to stimulate stem cell mobilization. G-CSF can disrupt the normal crosstalk between HSCs and the BM niche, resulting in stem cells egressing into the peripheral blood, where they can be harvested for transplantation. The majority of HSCs are located in a specialized niche within the BM, although some HSCs (∼1–5%) migrate into circulation. HSCs mobilization into the circulation is achieved through the administration of mobilizing agents. G-CSF or plerixafor are used to mobilize HSCs from the BM niche to the circulation and subsequent collection of HSCs. In fact, G-CSF or plerixafor is stem cell niche-directed compound used in the clinical setting for stem cell mobilization. The mobilization strategy is a basic mechanism for collecting stem cells from donor blood. Apart from the egress of HSCs from the niche into the blood, mobilization can also provide empty niches in the BM which increases the chance of donor cell engraftment (Lidonnici et al., 2017; Pahnke et al., 2019; Man et al., 2021). A similar approach is routinely employed to enhance the production of particular blood cell lineages in patients with leukocyte deficiencies. This can be mediated by the administration of lineage-specific hematopoietic cytokines such as granulocyte-macrophage colony-stimulating factor (GM-CSF) or erythropoietin are traditional hematopoietic stimulating factors (Ribatti and Tamma, 2019).

Since the depletion of resident hematopoietic stem and progenitor cells (HSPCs) is necessary for autologous and allogeneic HSCs transplantation, a niche-targeted strategy can be used for this purpose. Indeed, radiation and chemotherapeutics have been replaced by niche-targeted therapy to clear the HSC niche. The ideal condition is the presence of a target molecule on HSPC, not outside the hematopoietic system, such as CD117 (c-Kit) (Abadir et al., 2019). C-Kit and its ligand, SCF, are essential for hematopoiesis; however, they are also expressed outside the hematopoietic system, such as in the CNS, gastrointestinal tract, melanocytes, and epidermal cells (Manz and Russkamp, 2019). Therefore, early attempts to deplete HSPC using naked antibodies have shown limited efficacy. New approaches that use antibody combinations can be used to target niches. In this regard, antibody-drug conjugates and chimeric antibody receptor (CAR) T cells are novel modalities that efficiently deplete HSPC through targeted therapy (Abadir et al., 2019).

In addition to targeting niches using these approaches, researches has also focused on the *ex vivo* expansion of stem cells. Clinical transplantation of HSCs is limited and is influenced by several factors, such as the low number of isolated HSCs in patients. Allogeneic transplantation of HSCs has been applied clinically with a suitable donor; however, for patients without a matched donor, autologous HSCs have been used with a low risk of graft-versus-host (GVH). The limited number of HSCs that can be obtained from patients is a major challenge for clinical applications. As a result, there is an unmet medical need for HSCs expansion for applications in regenerative medicine and other therapeutic purposes. Niche signals play an essential role in regulating the self-renewal and differentiation of HSCs. Niche cells secrete various growth factors and cytokines to regulate the self-renewal and differentiation of HSCs (Zimran et al., 2021).

*Ex vivo* proliferation prior to transplantation is a potential solution for a low number of stem cells. For successful transplantation, ≥2.5 × 10^7^ cells/kg human body weight cells are needed. Stem cell expansion has gained much interest because of the importance of HSCs in the field of transplantation for the treatment of malignant and nonmalignant diseases. HSCs undergo symmetric and asymmetric modes of cell division, and the frequency of these divisions determines the number of stem cells and differentiated cells in the niche. Symmetric cell division contributes to the expansion of HSCs. To expand *ex vivo*, stem cells must divide and self-renew symmetrically without further differentiation (Tajer et al., 2019; Zimran et al., 2021).

Multiple strategies have been used to expand HSCs for transplantation. Co-culturing HSCs with MSCs can enhance HSCs expansion by producing secretory cytokines and facilitating cell-to-cell contact. It was found that co-culture of mesenchymal stromal cell-derived osteoblasts could increase the number of CD34^+^ HSCs by approximately 3.7-fold, which is mediated by the β-Catenin and Notch pathways (Michalicka et al., 2017).

Activation of the Notch signaling pathway can be applied for *ex vivo* expansion of HSPC, which is a conserved pathway in determining cell fate (Kumar and Geiger, 2017). Notch signaling is also important in development and hematopoiesis. Overexpression of Notch-related genes and their corresponding receptors increases self-renewal and the number of functional HSCs, and inhibits HSCs differentiation. Notch signaling is activated by interactions between Notch receptors and their ligands Delta and Jagged. This interaction promotes *ex vivo* expansion of HSCs (Lampreia et al., 2017). Dahlberg et al. used cytokine stimulation followed by activation of Notch signaling for *ex vivo* expansion of HSPC. If successful, this strategy may generate a high number of transplantable cells. Although *ex vivo* expansion strategies have been pursued for decades, they have largely failed to achieve significant success, which may be related to the complexity of the stem cell niche (Dahlberg et al., 2011). Furthermore, various growth factors and cytokines have been used for the *ex vivo* expansion of HSCs, including FGF, insulin-like growth factor (IGF), SCF, interleukin 6 (IL-6), TPO, and FMS-like tyrosine kinase 3 (Flt-3) ligand (Derakhshani et al., 2019).

As detailed above, niche-targeted interventions may be employed to support transplanted stem cells after stem cell transplantation. Compelling preclinical evidence suggests that activation of osteolineage cells by treatment with parathyroid hormone (PTH) can mediate the proliferation of nestin^+^ MSCs and stimulate osteolineage differentiation (Lawal, 2015). Esmaeili et al. evaluated the expansion of cord blood HSCs and HPCs on a mesenchymal cells as a feeder layer treated with PTH. *HES1* was overexpressed in the PTH-treated group compared with the feeder layer without PTH treatment. As an essential factor in HSC activation, this gene can expand cord blood-HSCs (Esmaeili et al., 2019). PTH treatment also contributes to the increase in some cytokines, such as GM-CSF, IL6, and C-kit, which are necessary for the proliferation and differentiation of HSCs (Sabbieti et al., 2017). PTH also plays a crucial role in bone remodeling. Several studies have reported that aging modulates the osteomicroenvironment and increases bone turnover, leading to bone loss. Age-related attenuation affects the osteogenic capacity of bone marrow MSCs. This reduced potential for osteogenecity correlates with increased adipogenesis (Kuo et al., 2017). Given the defective osteogenesis and decreased bone mass during aging, Jiang et al. investigated the effects of PTH on the osteogenic microenvironment in aged rats. According to their findings, PTH treatment enhanced the osteogenic potential of BM-MSCs and decreased adipogenesis in old rats (Jiang et al., 2018).

Furthermore, studies of skeletal MuSCs have indicated that their *ex vivo* expansion leads to a loss of regenerative potential. The MuSC function depends on the regulation of protein synthesis mediated by eIF2α phosphorylation. Therefore, preventing the dephosphorylation of eIF2α could promote *ex vivo* expansion of MuSCs concomitant with restoring regenerative capacity after engraftment into an animal model of muscular dystrophy (Lean et al., 2019). Kim et al. reported that the inhibition of aryl hydrocarbon receptor (AhR)-induced signaling can expand HSCs. CH223191, an ahR-selective antagonist, increased Lineage-CD34 + CD38-CD90 + CD45RA) HSCs, preserving the function of HSCs. CH223191 also increased the expansion of megakaryocyte lineage, indicating the numerical expansion of megakaryocyte lineage progeny (Kim et al., 2022).

### Niche-targeted approaches to promote regenerative activity

Compelling evidence suggests that multicellular organisms undergo a organ dysfunction as they age. The reason for this functional decline is the loss of stem cell number and activity over time. Alterations in the stem cell niche can activate stem cell suppressive mechanisms involved in tissue regeneration in several acute and degenerative diseases. This event is particularly apparent in physiological aging, as stem cell numbers and functions are profoundly altered in some tissues, resulting in reduced tissue function and delayed tissue repair (Wagers, 2012).

In the case of the blood system, the number of HSCs increases with age; however, this coincides with impaired functions. This phenomenon is manifested by decreased regenerative potential, impaired homing ability, reduction of cell polarity, shif from asymmetric to symmetric divisions, and myeloid skewing at the expense of lymphopoiesis. These alterations were initially attributed to the dysregulation of cell-intrinsic factors that may be responsible for reduced regenerative potential. However, recent evidence has revealed that the BM niche might affect HSCs aging (Ho and Méndez-Ferrer, 2020). This is supported by an elegant study in which the transplantation of old HSCs into young recipients led to reduced myeloid output compared with the transplantation of old HSCs into old recipients, indicating that old BM niches activate myeloid skewness. Rantes, an inflammatory cytokine, increases in the aging stem cell niche. Exposure of HSCs to Rantes leads to decreased levels of T cell progeny and increased myeloid progenitors. Likewise, animals lacking Rantes exhibit a reduced level of myeloid skewing in HSCs concomitant with the elevation of T cells and lymphoid-biased HSCs. This implies that niche factors play a critical role played in aged-related myeloid skewing (Ergen et al., 2012).

In line with this notion, Guidi et al. showed that osteopontin expression is reduced in murine BM. Osteopontin is produced by the BM stromal cells. In young mice, osteopontin regulates HSC pool size, homing ability, migration, and engraftment. The level of this glycoprotein is reduced in aged stroma and contributes to age-associated phenotypes in HSCs. Exposure of aged HSCs to thrombin can activate osteopontin, resulting in attenuation of HSC aging, indicating the critical role of osteopontin in ameliorating aging phenotypes in HSCs. Thus, niche can confer multiple aging-associated phenotypes on HSCs (Guidi et al., 2017). Another study provided evidence that an aged niche can restrain the rejuvenation of old HSCs, which is at least in part associated with reduced levels of osteopontin within aged niches (Guidi et al., 2021). Based on this information, an aged niche has a significant influence on the function of HSCs.

As illustrated in previous sections, MuSCs have a critical function in muscle regeneration. MuSCs gradually lose their functions during physiological and pathological aging. Several reports have demonstrated that the number of MuSCs declines with aging. Aged MuSC niches express FGF2, which causes MuSCs to escape quiescence and self-renewal loss. FGF2 activation partly contributes to the functional decline of MuSCs. Given the increased FGF-2 signaling during aging, inhibition of FGF signaling effectively reverses aging phenotype and restores self-renewal potential in aged MuSCs (Von Maltzahn, 2021). In addition, aged MuSC niches were found to have reduced levels of fibronectin, an ECM protein. Aged MuSCs exhibit impaired fibronectin-mediated signaling, resulting in MuSC dysfunction. Therefore, reconstituting fibronectin levels in the aged niche leads to the remobilization of stem cells as well as the restoration of youth-like regeneration in the muscle (Lukjanenko et al., 2016). Upon aging, niche-derived NF-κB signaling increases and disturbs MuSC function (García-García et al., 2021). It has also been reported that the administration of NF-B inhibitors can restore MuSC functionality (Oh et al., 2016). Moreover, the JAK/STAT pathway contributes to the maintenance of self-renewal ability in MuSCs; therefore, inhibition of this cascade restores age-associated declines in the regenerative potential of MuSCs (Sorensen et al., 2018; Chen et al., 2021).

A similar impairment in stem cell function was observed in the aged CNS. A growing body of evidence has demonstrated that aging negatively affects NSCs niches, ultimately influencing the regenerative potential in the CNS. Increasing age-related alterations are associated with a significant decrease in neurogenesis, coinciding with dynamic changes in the niche that impair normal homeostatic function (Nicaise et al., 2020). It is believed that these inexorable declines in stem cell function during aging contribute to chronic disease progression (Tchkonia and Kirkland, 2018). Parabiotic mouse models have shown that age-variant systemic factors, such as soluble factors and circulating HSCs, could alter the stem cell niche, which improves or reverses the aging-associated effects on the function of stem and progenitor cells (Ashapkin et al., 2020). This information emphasizes the dynamic nature of the stem cell niche and the possibility of endocrine and paracrine control of tissue regeneration. According to these data, distinct molecular entities can be used as niche-derived targets in therapeutic approaches. For example, skeletal muscle inactivates Notch signaling or hyperactivates Wnt or TGF-β signaling. Crosstalk between Notch and Wnt signaling pathways can maintain a balance between proliferation and differentiation. Activation of the Wnt signaling pathway in aged progenitors drives differentiation towards the fibrogenic cells instead of myogenic cells, resulting in tissue fibrosis and poor regeneration and muscle tissue repair (Blau et al., 2015). Therefore, niche-targeted strategies with the ability to restore the stem cell microenvironment to a more youthful milieu may be an effective strategy in reverting or ameliorating age-associated stem cell dysfunction. This can restore the regenerative capacity of the aging tissues. It is interesting to note that even young animals may undergo niche remodeling, which affects the regenerative potential of stem cells upon exposure to prolonged metabolic perturbations. Metabolic disorders, such as diabetes have been reported to significantly impair tissue repair, resulting in poor muscle repair, poor wound healing, and dysegulation of the immune system.

Similarly, blood-borne factors modulate regenerative defects in aged diabetic animals (Mashinchian et al., 2018). Additionally, recent studies on the hematopoietic system have revealed that diabetic mice and patients can effectively mobilize HSCs. Defective angiogenesis and aberrant inflammatory responses are common symptoms of aging and diabetes, both of which may have a common modification in stem cell niches for inhibiting tissue regeneration (Ferraro et al., 2011). Based on this evidence, niche-targeted therapeutics that suppress or reverse these alterations may benefit patients with diabetes and metabolic syndrome.

### Aging and cancer

In addition to aging and diabetes, niche-targeting interventions may also benefit cancer cases caused by deregulated cell growth. Increasing evidence indicates that a specialized tumor milieu plays a critical role in encouraging the growth of tumor cells and promoting metastatic behavior (Peinado et al., 2017). Tumor-derived stromal cells are often characterized by alterations in gene expression profiles, which may promote cell proliferation or inhibit normal differentiation (Von der Heide et al., 2017; Garcia-Gomez et al., 2018).

Several reports have shown profound deregulation of the bone morphogenetic protein (BMP) signaling pathways in cancer stem cells (CSCs), with major alterations in their receptors and downstream elements. The BMP pathway plays a vital role in the control of stem cells and their niches. BMP signaling regulates the proliferation and fate of normal HSCs and their interactions with their niches. Growing evidence has revealed that BMPs can promote cancer progression by orchestrating the interaction between cancer cells and their niche. These interactions direct the functions of tumor and stromal cells in the niche to promote the initiation of tumor growth, angiogenesis, local invasion, and metastasis. This leads to the establishment of cancer cells in the tumor niche (Sun et al., 2020). Extracellular antagonists such as chordin, noggin, follistatin, cerberus,g-1, and tolloid, can bind to BMPs and inhibit their interaction with corresponding receptors (Zylbersztejn et al., 2018). For example, Gremlin-1 plays a key role in the developmental stage and is responsible for cell proliferation *via* the suppression of BMP signaling. Accordingly, BMP antagonists may be a crucial components of the tumor stroma, creating an encouraging niche for cancer cell progression and survival (Sneddon et al., 2006).

Hedgehog signaling plays a critical role in the normal development and maintenance of homeostasis in adult tissues. In addition, this pathway modulates the microenvironment to provide a suitable niche for tumor cells by inducing angiogenesis, escaping from the immune system, and creating a niche for invasion and metastasis. Conversely, hedgehog downregulation is associated with age-related diseases (Hanna and Shevde, 2016). There is evidence that several solid tumors depend on Hedgehog signaling in their mesenchyme rather than in their tumor cells. The abnormal persistence of hedgehogs contributes to a variety of human cancers; thereby, the development of a novel approach directed toward the hedgehog pathway has gained much interest in cancer therapy (Salaritabar et al., 2019).

Recent studies have shown the significance of a CSC-like niche in promoting the production and maintenance of CSCs. Thus, the CSC niche plays a key role in maintaining the stem cell pool. CSCs are associated with tumor survival. These cells have high metastatic potential and give rise to solid tumor heterogeneity and complex architecture. CSCs express tumorigenic characteristics in response to signaling by their niches. Therapeutic approaches targeting pathways common in cancer stem cells and their niche may control tumor progression (Plaks et al., 2015).

A growing body of evidence indicates that aging is associated with cancer-initiating potential. DNA damage, telomere shortening, and metabolic reprogramming have been observed upon aging. Aged stem cells display telomere shortening, epigenetic remodeling, and replication stress, all of which are involved in the development of CSC-linked features. Notably, aging can enhance the CSC niche by altering the microenvironment to create an aged niche in the young niche (Fane and Weeraratna, 2020).

During the aging process, inflammatory responses are associated with the tumor microenvironment. Senescent microenvironments can induce alterations in cells with genomic instability to turn premalignant cells into cancer stem-like cells (Castro-Vega et al., 2015). Furthermore, aged fibroblasts can enhance tumor growth in several cancers. With increasing age, the stroma creates a pro-tumorigenic microenvironment, which is reflected by the secretory-associated specific phenotype (SASP), such as cytokines, chemokines, and energetic metabolites that act as the main drivers of age-related cancer initiation. Therefore, targeting the stroma is a potential therapy for age-related cancer (Elkhattouti et al., 2015). Moreover, senescence can create a metastatic niche, leading to elevated levels of inflammatory markers and matrix metalloproteinases (MMPs). Environmental remodeling provides a favorable environment for ovarian cancer metastasis (Harper et al., 2018).

The success of metastasis depends on the genetic and epigenetic deregulation of cancer cells and their interplay with the stromal microenvironment. The interaction between CSCs and their niche is bidirectional, in which cancer cells modify their niche; conversely, the niche acts as a “fertile soil” to produce all carcinoma cells of the tumor mass (Valcz et al., 2018).

Deregulated molecular pathways within the niche can influence the development, survival, migration, invasion, and chemoresistance of leukemia. Deregulated chemokine axes are seen within the niche of B-cell acute lymphoblastic leukemia, such as the overexpression of activin A. Activin A belongs to the TGFβ family and downregulates CXCL12 production by BM-MSCs. It also increased B-ALL cell migration *in vitro* in response to CXCL12. These molecular axes are associated with leukemic cells that communicate with the BM stromal niche. Targeting aberrant niches or their deregulated components may be beneficial for reversing the disease course (Dander et al., 2021). Myeloma and other cancer cells can hijack the BM niche and, in turn, can be modified by the niche. Therefore, targeting cancer niche interactions may be a promising therapeutic approach (Reagan and Rosen, 2016). Collectively, niche-directed strategies may also aid in the treatment of solid tumors. Targeted approaches result in the ablation of tumor-supporting niche cells, interference with homing to these niches, or disruption of niche ability to produce the required growth factors, thereby can be effective strategies for suppressing tumor growth and malignancy.

### Clonal hematopoiesis in aging

Clonal hematopoiesis of indeterminate potential (CHIP) is an age-associated condition resulting from the acquisition of somatic mutations. CHIP is a premalignant disorder that can be used as a predictor of hematological and cardiovascular diseases. However, during chronological aging, HSCs lose their ability to self-renew and exhibit skewed differentiation, which can increase the risk of anemia and impair adaptive and innate immunity. Most of these alterations are triggered by cell-intrinsic events such as genetic and epigenetic modifications, which determine the stages of clonal selection (Park and Bejar, 2018; Mondor et al., 2019).

Although previous studies in mice have suggested that HSC aging is largely a result of intrinsic molecular changes within cells, the significance of microenvironment-related factors may be undervalued in short-lived models with limited exposure to environmental stressors.

In humans, the BM niche plays a prominent role in hematopoiesis. Several lines of evidence indicate that external pressure (e.g., chronic inflammation) can influence normal hematopoiesis. Despite an understanding of the genetic mechanisms of clonal hematopoiesis in malignant states, the clinical importance of clonality in healthy aging remains unclear. As part of this review, we briefly discuss the cell-intrinsic molecular mechanisms involved in the aging of HSCs and describe the clonal drivers associated with aging hematopoiesis. In this section, we discuss various cell-extrinsic factors that potentially influence CHIP development within the BM niche.

### Hematopoietic stem cell aging: cell-intrinsic mechanisms

The transplantation of aged HSCs into young mice retains the aging phenotype, suggesting that intrinsic changes are the primary cause of HSC aging. Several cell-intrinsic mechanisms implicated in the aging of HSCs, which may be related to clonal selection during aging, are described below (Dykstra et al., 2011).

### DNA damage

It has been proposed that accumulation of DNA damage is an irreversible cause of HSC aging. Mutations in genes encoding DNA damage response (DDR) proteins may trigger senescence of HSCs, leading to depletion of the functional pool. This theory is supported by increased levels of histone 2A family member X (γ-H2AX) and foci formation in aged HSCs. γ-H2AX is a sensitive indicator for DNA double-strand breaks (DSB). HSC reserves are preserved with age in mice lacking DNA repair genes; however, HSCs exhibit severe functional limitations. Similarly, HSCs in mice lacking the BRCA1 gene showed expansion in the BM, but exhibited less hematopoietic colony formation *in vitro*. Furthermore, somatic mutations in cancer-associated genes may facilitate clonal expansion through selective growth. It is important to note that these paths to clonal hematopoiesis are not equally exclusive and each is influenced by external factors. However, these pathways also have different clinical implications (Vasanthakumar et al., 2016).

### Telomere attrition

Aging of HSCs is associated with telomere erosion, a specific form of DNA damage. A sequence of repetitive DNA located at the ends of chromosomes prevents DDR activation. Each cell division results in shorter telomeres, which ultimately cause unprotected chromosome ends, eliciting senescence or apoptosis. By breeding telomerase mRNA-deficient mice, telomeres are eventually critically shortened, resulting in impaired long-term viability of cells and compromised renewal of HSCs. Stochastic loss of HSCs occurs through telomere attrition and expansion of clones with superior growth characteristics. According to two studies of germline sequence variants in the TERT locus, telomerase activity may play a substantial role in the development of clonal hematopoiesis. TERT and TERC are components of the telomere complex that are altered in telomeropathies such as dyskeratosis congenita. There is a high probability that short telomere syndromes cause early BM failure in children, and a higher propensity for MDS or acute myeloid leukemia (AML) in adults. Accelerated telomere erosion resulting from underlying genetic mutations, in combination with cytokine overproduction, may promote depletion of the HSC pool and premalignant clonal expansion (Hinds et al., 2016).

## Epigenetics

Clonal mutations associated with aging primarily involve DNA methyltransferase 3A (DNMT3A) and Tet Methylcytosine Dioxygenase 2 (TET2), two genes responsible for the DNA methylation/demethylation status. Therefore, the maintenance of epigenetic regulation is crucial for proper regulation of HSCs. The methylation profiles of young and old human HSCs showed hypomethylation of genes that were ordinarily hypomethylated and activated during the differentiation of myeloid cells. Such alterations in the epigenomic profile during HSC aging may explain the lower differentiation potential of older HSCs. The higher replicative history associated with aging appears to be partially responsible for the decreased self-renewal capacity of the HSCs. Inaccuracy in transmitting epigenetic marks to daughter cells can gradually and randomly cause erosion of epigenetic elements during HSC division with age. Aged HSCs may exhibit aberrant epigenetic status due to the loss of transcriptional fidelity. The inability of daughter cells to maintain the HSC pool may pave the way for further clonal selection. This process can be accelerated by the presence of somatically mutated epigenetic regulators (Beerman et al., 2013).

Is clonal hematopoiesis a normal result of aging?

In the presence of mutations in genes associated with myeloid malignancy, it is important to determine whether CHIP is specifically indicative of preleukemic conditions.

Several seminal studies have shown that CHIP is associated with an increased risk of hematologic malignancies, similar to monoclonal gammopathies and B cell lymphocytosis. Based on two population-based studies, it appears that individuals without antecedent candidate gene mutations have a negligible risk of developing hematological malignancies. Approximately 0.5% of the 12,000 participants evaluated in both studies developed hematological malignancies, and only one-third had candidate driver mutations. There was an association between clonal abundance and cancer risk, suggesting that clone size was a more relevant factor than mere presence. With error-corrected sequencing, researchers can detect somatic variants at a level two orders of magnitude lower than that of standard next-generation sequencing (NGS). Using this ultrasensitive technique, Young et al. studied the prevalence of clonal hematopoiesis in healthy middle-aged women (Young et al., 2016).

Clonal hematopoiesis was observed in 95% of the studied individuals, often with mutations in DNMT3A and TET2. Using a whole-genome approach, Zink et al. discovered that elderly individuals exhibit macroscopic clonal hematopoiesis.

A previous study found that only 5% of middle-aged individuals had clonal hematopoiesis; therefore, clonal hematopoiesis is not necessarily pathogenic but rather a nearly universal feature of aging. In advanced age, when most of HSC proliferative capacity have diminished, clonal expansion may be a compensatory mechanism to maintain normal hematopoiesis. Experiments in murine models have demonstrated that the two most prevalent mutations associated with clonal hematopoiesis, DNMT3A and TET2, promote the self-renewal of HSCs. However, mouse models do not necessarily reflect CHIP in humans. The phenotypic profile of TET2-deficient mice is similar to that of chronic myelomonocytic leukemia in humans. As an example of how mutations can bestow HSCs with adequate self-renewal potential, an 115-year-old woman with a single clone harboring 450 somatic mutations maintained normal blood production during her prolonged life. She died from metastatic gastric cancer rather than any malignant hematopoietic disorder (Holstege et al., 2014). Although this case presents a highly mutated clone, there is still much to learn about clonal hematopoiesis and the risk of malignant transformation.

## Conclusion

Over the past years, the advances of stem cell research has revealed many aspects of stem cell niches, including stem cell interactions and other niche cells as well as the ECM, signaling pathways (e.g., Wnt, Notch, BMP), various soluble factors, biochemical and biophysical components of stem cell niche. Although stem cells hold tremendous promise in regenerative medicine, they do not play a role in tissue hemostasis and regeneration. The interaction of stem cells with adjacent stem cells and the surrounding ECM microenvironment can determine the migration, proliferation, differentiation and regenerative capacity of stem cells. Since stem cells are extremely responsive to extrinsic signals, they must interact closely with their environment to receive niche signals, which drive their function.

## Future directions

There is now an increased interest in investigating the BM niche owing to its therapeutic and translational potential. Various approaches, including humanized niche, model single-cell profiling, and spatial transcriptomics data, will contribute to consolidating and deepening our understanding of how the BM niche can support HSC function over time. It is generally accepted that the intrinsic elements driving HSC aging are mostly fixed within the cells, and microenvironment or systemic rejuvenation interventions have little effect on this condition (166). It is interesting to note that rejuvenating aged HSCs is beneficial to different tissues and may also impact the BM niche. Currently, we are only beginning to explore the boundaries between the intrinsic and extrinsic aging of HSCs and how they are interconnected. Considering the current perspective, interventions affecting both features might profoundly affect hematopoiesis. Ideally, this combined strategy targeting HSC intrinsic and extrinsic aging could be applied to other somatic stem cells and tissues to eventually extend the lifespan and slow the aging process in the entire body.

In this regard, a better understanding of stem cells, the surrounding microenvironment, and molecular signals may help develop niche-targeted interventions to promote tissue regeneration and reverse or ameliorate age-related complications. Accordingly, by targeting deregulated molecular pathways or dysfunctional components within the niches, novel strategies can be developed to combat stem cell loss, such as that caused by aging or degenerative diseases. In addition to aging, targeting aberrant niches or their deregulated component may be beneficial in preventing or reversing the malignant transformation in cancers).
